# A Vasopressin-Induced Change in Prostaglandin Receptor Subtype Expression Explains the Differential Effect of PGE_2_ on AQP2 Expression

**DOI:** 10.3389/fphys.2021.787598

**Published:** 2022-01-21

**Authors:** Peter M. T. Deen, Michelle Boone, Horst Schweer, Emma T. B. Olesen, Claudia Carmone, Jack F. M. Wetzels, Robert A. Fenton, Marleen L. A. Kortenoeven

**Affiliations:** ^1^Department of Physiology, Radboud University Nijmegen Medical Center, Nijmegen, Netherlands; ^2^Department of Pediatrics, Philipps-University Marburg, Marburg, Germany; ^3^Department of Biomedicine, Aarhus University, Aarhus, Denmark; ^4^Department of Biomedical Sciences, University of Copenhagen, Copenhagen, Denmark; ^5^Department of Endocrinology and Nephrology, North Zealand Hospital, Hillerød, Denmark; ^6^Department of Nephrology, Radboud University Nijmegen Medical Center, Nijmegen, Netherlands; ^7^Department of Cardiovascular and Renal Research, Institute of Molecular Medicine, University of Southern Denmark, Odense, Denmark

**Keywords:** water transport, AQP2, vasopressin, prostaglandin, mpkCCD, PGE2, EP1, EP4

## Abstract

Arginine vasopressin (AVP) stimulates the concentration of renal urine by increasing the principal cell expression of aquaporin-2 (AQP2) water channels. Prostaglandin E_2_ (PGE_2_) and prostaglandin_2α_ (PGF_2α_) increase the water absorption of the principal cell without AVP, but PGE_2_ decreases it in the presence of AVP. The underlying mechanism of this paradoxical response was investigated here. Mouse cortical collecting duct (mkpCCD_c14_) cells mimic principal cells as they endogenously express AQP2 in response to AVP. PGE_2_ increased AQP2 abundance without desmopressin (dDAVP), while in the presence of dDAVP, PGE_2_, and PGF_2α_ reduced AQP2 abundance. dDAVP increased the cellular PGD_2_ and PGE_2_ release and decreased the PGF_2α_ release. MpkCCD cells expressed mRNAs for the receptors of PGE_2_ (EP1/EP4), PGF_2_ (FP), and TxB_2_ (TP). Incubation with dDAVP increased the expression of EP1 and FP but decreased the expression of EP4. In the absence of dDAVP, incubation of mpkCCD cells with an EP4, but not EP1/3, agonist increased AQP2 abundance, and the PGE_2_-induced increase in AQP2 was blocked with an EP4 antagonist. Moreover, in the presence of dDAVP, an EP1/3, but not EP4, agonist decreased the AQP2 abundance, and the addition of EP1 antagonists prevented the PGE_2_-mediated downregulation of AQP2. Our study shows that in mpkCCD_c14_ cells, reduced EP4 receptor and increased EP1/FP receptor expression by dDAVP explains the differential effects of PGE_2_ and PGF_2α_ on AQP2 abundance with or without dDAVP. As the V2R and EP4 receptor, but not the EP1 and FP receptor, can couple to Gs and stimulate the cyclic adenosine monophosphate (cAMP) pathway, our data support a view that cells can desensitize themselves for receptors activating the same pathway and sensitize themselves for receptors of alternative pathways.

## Introduction

To prevent dehydration, an adequate maintenance of water homeostasis is essential. In this process, the kidney plays a critical role. In response to hypernatremia or hypovolemia, arginine vasopressin (AVP) is released from the posterior pituitary gland. Subsequently, binding of AVP to the basolateral vasopressin type-2 receptor (V2R) in the connecting tubule and collecting duct principal cells in the kidney results in the redistribution of aquaporin-2 (AQP2) water channels from intracellular vesicles to the apical membrane, greatly increasing the osmotic water permeability, a prerequisite for forming concentrated urine (Knepper, 1997). In addition, AVP also increases the expression of AQP2 *via* phosphorylation of the cyclic adenosine monophosphate (cAMP)-responsive element binding protein, which activates transcription from the AQP2 promoter (Terris et al., 1996; Matsumura et al., 1997; Yasui et al., 1997).

**Figure 1:**
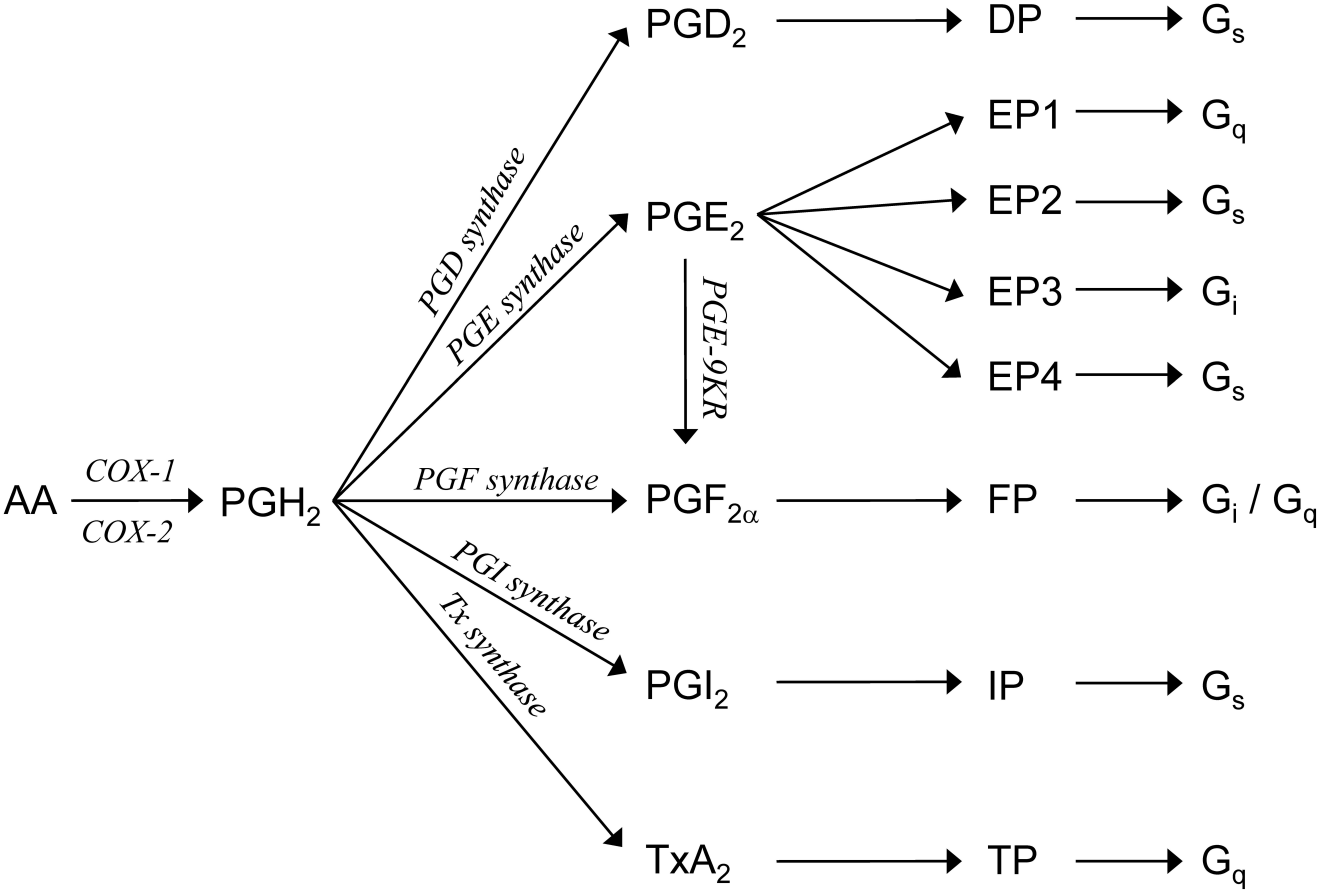
Prostaglandin synthesis. Arachidonic acid (AA) is metabolized by COX1 or COX2 to PGH_2_. PGH_2_ is enzymatically converted, by specific synthases [prostaglandin D (PGD) synthase, prostaglandin E (PGE) synthase, prostaglandin F (PGF) synthase, prostaglandin I (PGI) synthase, and thromboxane synthase] or prostaglandin E 9-ketoreductase (PGE-9KR), to one of five primary prostanoids, namely, PGI_2_, PGD_2_, PGE_2_, PGF_2α_, or TxA_2_. Each prostanoid interacts with distinct members of a subfamily of the G-protein-coupled receptors. PGI_2_ activates the IP receptor, PGD_2_ activates the DP receptor, PGF_2α_ activates the FP receptor, and TxA_2_ activates the TP receptor. PGE_2_ interacts with one of four distinct EP receptors.

Besides AVP, several other signaling molecules regulate the water balance by antagonizing the AVP-induced water transport (Boone and Deen, 2008). One such group of molecules is the prostaglandins (Figure 1). Prostaglandins can bind to their unique G-protein-coupled receptors (i.e., DP, FP, IP, and TP) or to one or more of four different PGE_2_ receptors (i.e., EP1, EP2, EP3, and EP4). Some of these receptors (i.e., DP, EP2, EP4, and IP) are Gs-coupled and thus increase intracellular cAMP levels when activated, whereas others are coupled to Gi (i.e., EP3 and FP), reducing the cAMP synthesis, and/or Gq (i.e., EP1, FP, and TP), inducing calcium mobilization (Breyer et al., 1998; Hebert et al., 2005; Hao and Breyer, 2008).

Of the different prostaglandins, PGE_2_ in particular has been shown to decrease AVP-stimulated water reabsorption in perfused collecting ducts (Hebert et al., 1990; Nadler et al., 1992; Sakairi et al., 1995). In addition, PGE_2_ is also involved in the pathological regulation of water reabsorption. PGE_2_ has been suggested to play an important role in the development of lithium-induced nephrogenic diabetes insipidus (NDI). This is based on the observation that the renal expression of the enzyme cyclooxygenase 2 (COX-2), involved in prostaglandin production, is markedly increased in lithium-treated mice, resulting in an increased excretion of urinary PGE_2_ (Rao et al., 2005). Also, treatment with a COX-2 inhibitor alleviated lithium-induced polyuria (Kim et al., 2008). Similarly, in the bilateral ureteral obstruction, associated with AQP2 downregulation, COX-2 protein abundance as well as the concentrations of PGE_2_ and other prostanoids are increased in the kidney inner medulla (Norregaard et al., 2010). Administration of COX-2 inhibitor prevents the increase of urinary PGE_2_ and the downregulation of AQP2 in inner medullary collecting ducts seen after the bilateral ureteral obstruction (Norregaard et al., 2005). In addition, PGE_2_ has recently been suggested to be instrumental in the increased free water reabsorption and volume expansion, leading to thiazide-induced hyponatremia (Ware et al., 2017). Besides PGE_2_, PGF_2α_ can also inhibit AVP-stimulated water permeability in the collecting duct (Zook and Strandhoy, 1981; Hebert et al., 2005).

Paradoxically, PGE_2_ increases the osmotic water permeability in the absence of AVP (Hebert et al., 1990; Sakairi et al., 1995). The underlying mechanism of this switch in function, however, is still unclear. Therefore, in the present study, we utilized the cortical collecting duct (mpkCCD_c14_) cells of a mouse as a model system for the renal principal cell to delineate how prostaglandins can exert their diverse effects on the principal cell water reabsorption in the presence or absence of AVP.

## Materials and Methods

### Cell Culture

Mouse mpkCCD_c14_ cells were maintained essentially as described (Hasler et al., 2002). Cells were seeded at a density of 1.5 × 10^5^ cells/cm^2^ on semipermeable filters (Transwell®, 0.4 μm pore size, Corning Costar, Cambridge, MA) and cultured for 8 days. Unless stated otherwise, the cells were exposed to 1 nM of the V2R agonist desmopressin (dDAVP) at the basolateral side during the last 96 h, to maximally induce the AQP2 expression (Li et al., 2006). Cells were incubated with 10 μM indomethacin, 1 μM PGE_2_ (both Sigma, St. Louis, MO, USA), 1 μM PGF_2α_ (Calbiochem, San Diego, CA), 300 nM of EP1/EP3 agonists sulprostone (Sigma, St. Louis, MO, USA), 1 μM of EP4 agonists CAY10580, 0.5 μM of EP4 antagonist Gw627368, 2.5 nM of the EP4 antagonist L161982, 20 μM of EP1 antagonist Sc-51089, or 100 nM of EP1 antagonist Ono-8711 (all Cayman Chemical, Ann Arbor, Michigan, USA) during the last 48 h. The medium was replaced after 24 h, or in experiments using the EP agonists or antagonists, the medium was replaced every 12 h.

### Immunoblotting

MpkCCD_c14_ cells grown on 1.13 cm^2^ filters were lysed using 200 μl Laemmli. Sodium dodecyl sulfate-polyacrylamide gel electrophoresis, blotting, and blocking of the polyvinylidene fluoride membranes were carried out as described previously (Kamsteeg et al., 1999). Membranes were incubated for 16 h with 1:3,000-diluted affinity-purified rabbit anti-AQP2 antibodies [R7 (Deen et al., 1994) or Novus Biologicals, Littleton, CO] in Tris-buffered saline Tween-20 (TBS-T) supplemented with 1% w/v nonfat dried milk. Blots were incubated for 1 h with 1:5,000-diluted goat anti-rabbit IgGs (Sigma, St. Louis, MO) as secondary antibodies coupled to horseradish peroxidase. Proteins were visualized using enhanced chemiluminescence (ECL, Pierce, Rockford, IL).

### (Quantitative) Reverse-Transcriptase Polymerase Chain Reaction

MpkCCD_c14_ cells were grown as described above, and total RNA was isolated using the TriZol extraction reagent (Gibco, Life Technologies, Rockville, MD), according to the instructions of the manufacturer. To remove genomic DNA, total RNA was treated with DNase (Promega, Madison, WI) for 1 h at 37°C, extracted with phenol/chloroform, and precipitated. RNA was reverse-transcribed into cDNA using Moloney Murine Leukemia Virus reverse-transcriptase and random primers (Promega, Madison, WI). During cDNA production, a control reaction without the reverse-transcriptase enzyme was conducted to exclude genomic DNA amplification. Exon overlapping primers were designed for prostaglandin receptors (see Table 1). Amplification was performed using the cDNA equivalent of 5 ng RNA for 40 cycles (i.e., 95°C 45 s, 50°C 1 min, and 72°C 1.5 min). β-actin was used as a positive control for cDNA amplification. cDNA from the tissue reported to express the particular receptor was taken along as a positive control. The proper identity of products was confirmed using the restriction analysis.

**Table 1:** Primer sequences.

**Protein**	**Forward primer (5'-3')**	**Reverse primer (5'-3')**	**Product size (bp)**
DP	AGGAGCTGGACCACTTTGTG	TCACAGACAGGAAACGCAAG	159
EP1	GCACGGAGCCGAGGAGC	GCAGGGGCTCATATCAGTGG	107
EP2	TCGCCATATGCTCCTTGC	TCCTCTGACACTTTCCACAAA	449
EP3	GCAGAATCACCACGGAGACG	GCGAAGCCAGGCGAACTG	190
EP4	TACGCCGCCTTCTCTTACAT	TTCACCACGTTTGGCTGATA	380
FP	CGTCACGGGAGTCACACTCT	TTCACAGGTCACTGGGGAAT	190
IP	CATGACCGTCATCATGGCCGTG	GTTGAAGGCGTTGAAGCGGAAGG	120
TP	GTGGGCATCATGGTGGTGG	CACACGCAGGTAGATGAGCAGC	168
β actin	GTATGCCTCTGGTCGTACCAC	ACGATTTCCCTCTCAGCTGTG	201
18S	GTAACCCGTTGAACCCCATT	CCATCCAATCGGTAGTAGCG	151

SYBR Green real-time quantitative reverse-transcriptase polymerase chain reaction (RT-PCR) was performed on an iQ5 Real-Time PCR Detection System from Bio-Rad by utilizing the SYBR Green PCR Master Mix (Applied Biosystems Foster City, CA). Signals for the ribosomal 18S were used to normalize for differences in the amount of starting cDNA.

### Prostanoid Analysis

Samples were prepared as described previously (Schweer et al., 1994) with minor modifications. In brief, cell culture supernatants were spiked with ~1 ng of deuterated internal standards, and the methoximes were obtained through the reaction with an O-methylhydroxylamine hydrochloride-acetate buffer. After acidification to pH 3.5, prostanoid derivatives were extracted, and the pentafluorobenzylesters were formed. Samples were purified by thin layer chromatography, and a broad zone with R_F_ 0.03–0.4 was eluted. After withdrawal of the organic layer, trimethylsilyl ethers were prepared by the reaction with bis(trimethylsilyl)-trifluoroacetamide and thereafter, subjected to the gas chromatography-tandem mass spectrometry (GC/MS/MS) analysis on a Finnigan MAT TSQ700 GC/MS/MS (Thermo Electron Corp., Dreieich, Germany) equipped with a Varian 3400 gas chromatograph (Palo Alto, CA) and a CTC A200S autosampler (CTC Analytics, Zwingen, Switzerland).

### Statistical Analysis

Student's *t*-test was applied to compare two groups with Gaussian distribution. Comparisons of more than two groups were performed using a one-way ANOVA followed by a Dunnett multiple comparison test. Levene's test was used to compare variances. *P*-values < 0.05 were considered significant. Immunoblotting signals were analyzed using the Bio-Rad software. Data are presented as mean ± standard error of the mean (SEM).

## Results

### In MpkCCD Cells, Regulation of AQP2 Expression by Prostanoids Is Modulated by AVP

**Figure 2:**
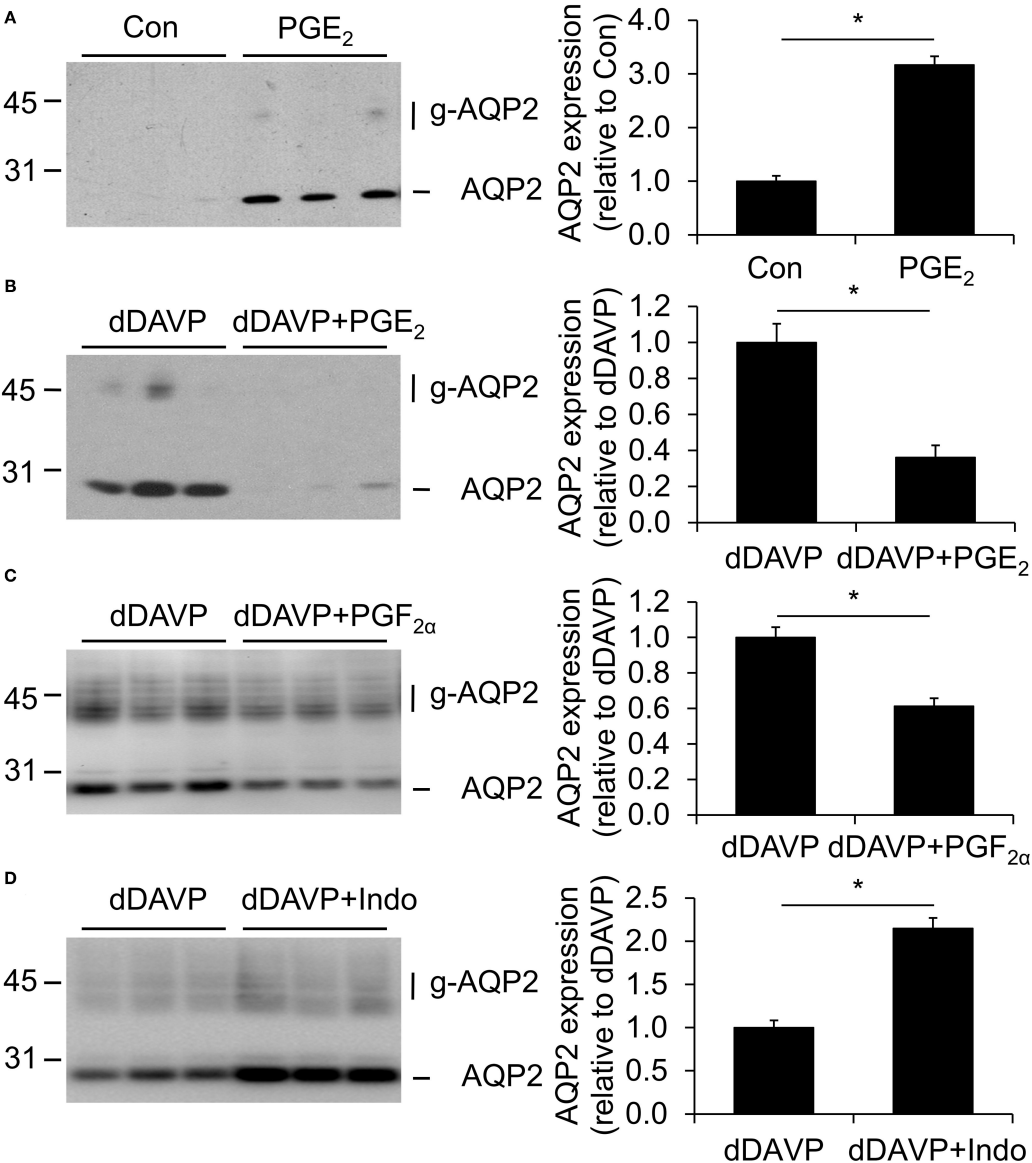
Effect of prostaglandins on aquaporin-2 (AQP2) expression. MpkCCD_c14_ cells were grown for 8 days, either with or without 1 nM desmopressin (dDAVP) stimulation for the last 4 days and with or without 1 μM PGE_2_ **(A,B)**, 1 μM PGF_2α_ **(C)**, or 10 μM indomethacin **(D)** during the last 48 h. Cells were lysed and subjected to immunoblotting for AQP2. Molecular masses (in kDa) are indicated on the left. Nonglycosylated (AQP2) and complex-glycosylated (g-AQP2) forms of AQP2 are detected and densitometrically quantified. Significant differences from control or dDAVP alone (*p* < 0.05) are indicated by an asterisk. Bars are mean values of nine filters per condition (±SEM).

To analyze the effect of PGE_2_ on the AQP2 expression, mpkCCD_c14_ cells were grown to confluence for 8 days, either with or without 1 nM of the V2R agonist dDAVP for the last 4 days and with or without 1 μM PGE_2_ during the last 48 h. PGE_2_ increased the AQP2 abundance in the absence of dDAVP but decreased it in the presence of dDAVP (Figure 2). In the presence of dDAVP, 1 μM PGF_2α_ also decreased the AQP2 abundance.

To test whether COX inhibition affects the dDAVP-induced AQP2 expression, cells were grown as described above, i.e., the last 4 days in the presence of dDAVP and the last 48 h in the presence of 10 μM indomethacin. Subsequent immunoblotting showed an increased AQP2 abundance with indomethacin (Figure 2), suggesting that dDAVP-treated mpkCCD_c14_ cells produce prostanoids, which decrease the AQP2 abundance.

### dDAVP Changes Prostanoid Production in MpkCCD Cells

**Figure 3:**
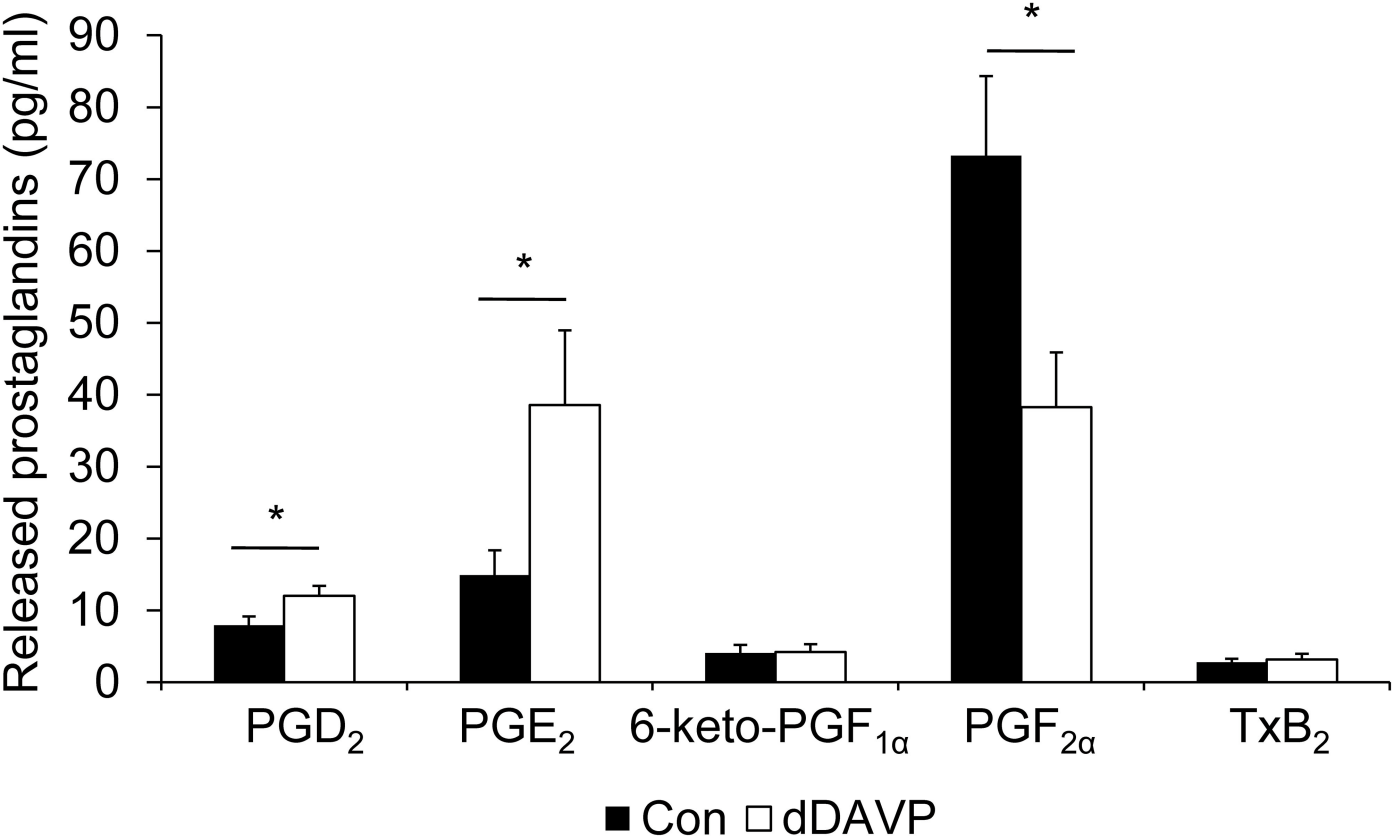
Effect of dDAVP on prostaglandin production. MpkCCD_c14_ cells were grown for 8 days and treated with or without (con) 1 nM dDAVP for the last 4 days. The medium from both sides, incubated with the cells for 24 h, was collected, and prostaglandin concentrations were determined. Bars are mean values of eight filters per condition (±SEM). Significant differences from control (*p* < 0.05) are indicated by an asterisk.

To determine whether mpkCCD_c14_ cells produce PGE_2_ or other prostanoids, and whether the presence of dDAVP affects the release of these prostanoids, cells were grown as above, i.e., with or without dDAVP for the last 4 days, after which the medium was collected and analyzed for the presence of prostanoids. Prostaglandin concentrations from the fresh medium were subtracted. The major prostanoids released from control cells were PGE_2_ and PGF_2α_, while levels of PGD_2_, 6-keto-PGF_1α_ (i.e., a stable metabolite of PGI_2_), and TxB_2_ (i.e., a stable metabolite of TxA_2_) were lower and bordering on their detection limit (Figure 3). The dDAVP treatment significantly increased the production of PGD_2_ and PGE_2_, while PGF_2_α levels were decreased. No effect of dDAVP was observed on the release of 6-keto-PGF_1α_ or TxB_2_.

### dDAVP Differentially Affects Prostanoid Receptor mRNA Expression in MpkCCD Cells

The effects of prostaglandins on the AQP2 expression are conferred by effects on their respective receptors. Immunoblotting was unsuitable to examine the expression of the individual prostaglandin receptors (not shown). Therefore, we determined the mRNA expression of the prostaglandin receptors in mpkCCD cells using the RT-PCR.

**Figure 4:**
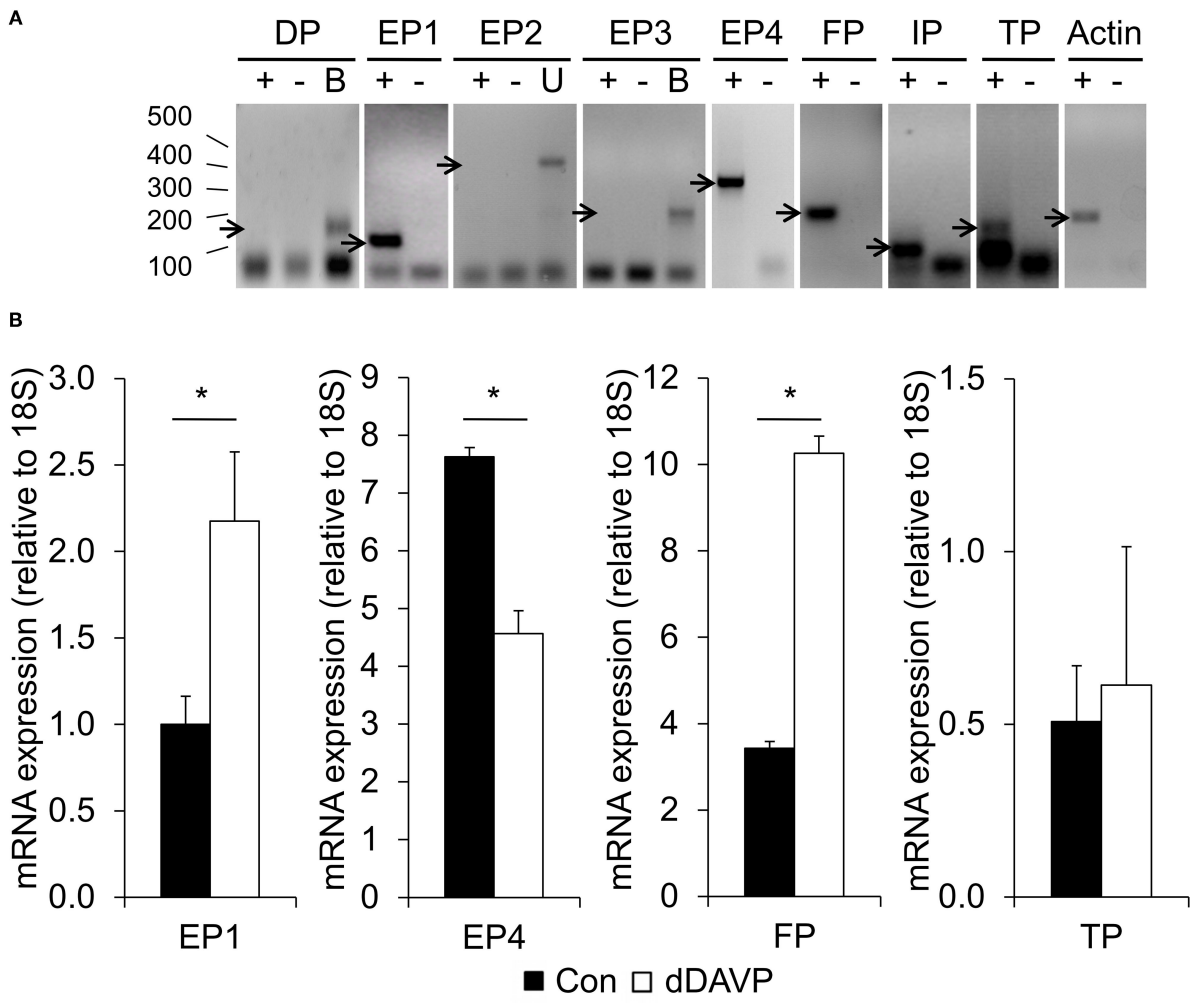
Prostaglandin receptor expression. **(A)** MpkCCD_c14_ cells were grown for 8 days. Cells were lysed, total RNA was isolated, and RNA was reverse-transcribed into cDNA. By using the reverse-transcriptase polymerase chain reaction (RT-PCR), the expression of the prostaglandin receptors was analyzed. β-actin was used as a positive control for cDNA amplification. ± = with or without reverse transcriptase during the cDNA production. B, brain, U, uterus. Sizes in bp are indicated on the left. Arrows point at product of expected size. **(B)** MpkCCD_c14_ cells were grown for 8 days and incubated with or without (con) 1 nM dDAVP for the last 4 days. Total RNA was isolated, RNA was reverse-transcribed into cDNA, and the relative expression of the prostaglandin receptors was analyzed by performing the quantitative (q)RT-PCR. The signals obtained from the house-keeping 18S were used to normalize for difference in the amount of starting cDNA. Bars are mean values of eight filters per condition (±SEM). Significant differences (*p* < 0.05) from control are indicated by an asterisk.

From unstimulated cells, cDNA products of the expected size were obtained for EP1, EP4, FP, and TP receptors (Figure 4A). While PCR products for EP2, EP3, or DP receptors were found in control tissues, no products were obtained in mpkCCD_c14_ cells, indicating that these receptors are not expressed. A detectable expression of the IP receptor was inconsistent. The same receptors were expressed in mpkCCD cells treated with dDAVP (not shown).

To test if the levels of the expressed prostanoid receptors were influenced by dDAVP, we determined their relative expression by using the qRT-PCR. dDAVP increased the expression of the EP1 and FP receptor, while the expression of the EP4 receptor was significantly decreased (Figure 4B). No difference was detected in the expression of the TP receptor.

### Modulation of PGE_2_ Receptor Subtype Expression by dDAVP Explains the Differential Effect of Prostanoids on AQP2 Abundance

As the EP1/FP receptors and EP4 receptors are coupled to Gi/Gq and Gs (Figure 1), respectively, an altered activation of these receptors due to their changes in the expression with dDAVP could explain the differential effect of prostanoids on the AQP2 abundance. To further explore the roles of the different PGE_2_ receptor subtypes in mediating the effects of PGE_2_ on AQP2 levels, we used EP receptor-specific agonists and antagonists.

**Figure 5:**
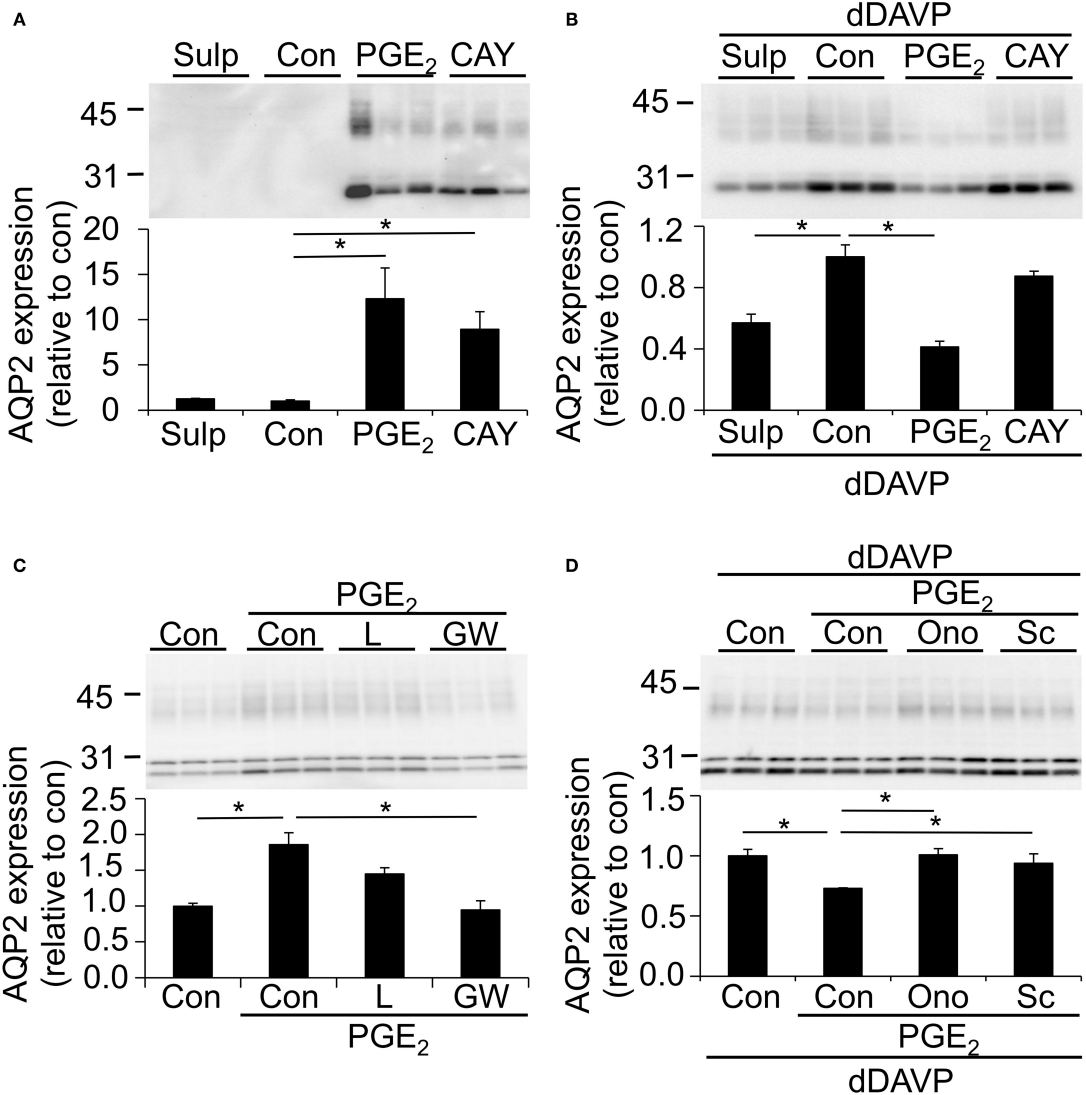
Effect of different PGE_2_ receptor agonists and antagonists on the AQP2 expression. MpkCCD_c14_ cells were grown for 8 days, either with our without 1 nM dDAVP stimulation for the last 4 days and with or without 1 μM PGE_2_ during the last 48 h. **(A,B)** Cells were incubated with1 μM sulprostone (Sulp) or 300 nM Cayman10580 (Cay) during the last 48 h. **(C)** Cells were incubated with 0.5 μM of EP4 antagonist Gw627368 (GW) or 2.5 nM of the EP4 antagonist L161982 (L) during the last 48 h. **(D)** Cells were incubated with 20 μM of EP1 antagonist Sc-51089 (Sc) or 100 nM of EP1 antagonist Ono-8711 (Ono) during the last 48 h. Cells were lysed and subjected to immunoblotting for AQP2. Molecular masses (in kDa) are indicated on the left. Nonglycosylated AQP2 (29 kDa) and complex-glycosylated (40–45 kDa) forms of AQP2 are detected and densitometrically quantified. Significant differences from control (con, *p* < 0.05) are indicated by an asterisk. Bars are mean values of 9 **(A,B)** or 6 **(C,D)** filters per condition (±SEM).

MpkCCD cells were grown as described above, i.e., stimulated with or without dDAVP, and incubated with the EP4 agonist CAY10580 or the EP1/EP3 agonist sulprostone (Kiriyama et al., 1997; Billot et al., 2003) during the last 48 h. As the EP3 receptor is not expressed in mpkCCD cells (Figure 4A), sulprostone will act as a specific EP1 agonist in these cells. Consistent with a contribution of EP4 to the prostanoid-stimulated AQP2 abundance in unstimulated cells, CAY10580 and PGE_2_ increased the AQP2 abundance as compared with unstimulated cells or cells incubated with sulprostone (Figure 5A). In cells stimulated with dDAVP, however, CAY10580 did not affect the AQP2 abundance, while both sulprostone and PGE_2_ decreased the AQP2 abundance, therewith, illustrating an important contribution of the EP1 receptor in reducing the AQP2 abundance in dDAVP-stimulated mpkCCD, cells (Figure 5B).

To further investigate the role of the EP4 receptor in the prostanoid-induced AQP2 abundance, mpkCCD cells were treated with PGE_2_ with or without the EP4 antagonists L161982 and Gw627368. While PGE_2_ alone again increased the AQP2 abundance significantly, Gw627368 completely blocked the PGE_2_-mediated AQP2 increase, whereas L161982 had a tendency to decrease the AQP2 expression relative to cells treated with PGE_2_ alone (Figure 5C).

To investigate the role of EP1 in the PGE_2_-mediated AQP2 decrease in dDAVP-treated cells, mpkCCD cells were stimulated with dDAVP and incubated with or without PGE_2_ and the specific EP1 antagonists Sc-51089 or Ono-8711. Both antagonists fully prevented the PGE_2_-mediated downregulation of AQP2 (Figure 5D), illustrating an important contribution of the EP1 receptor in the regulation of AQP2.

## Discussion

### Prostanoids Affect AQP2 Expression in MpkCCD Cells

Prostaglandin E_2_ reduce the AVP-stimulated water reabsorption in the collecting duct (Hebert et al., 1990; Nadler et al., 1992), while in the absence of AVP, *ex vivo* water permeability is increased by PGE_2_ (Sakairi et al., 1995). A short-term action of PGE_2_ is to alter the localization of AQP2 at the plasma membrane (Zelenina et al., 2000; Nejsum et al., 2005; Olesen et al., 2011). Here, we showed that long-term PGE_2_ affects the abundance of the AQP2 protein. PGE_2_ attenuated the dDAVP-induced AQP2 expression, while PGE_2_ stimulated the AQP2 abundance in the absence of dDAVP. In addition, dDAVP-stimulated AQP2 levels were decreased after the application of PGF_2α_, explaining the inhibition of water reabsorption in the collecting duct observed after the PGF_2α_ treatment (Zook and Strandhoy, 1981; Hebert et al., 2005). Furthermore, blocking the prostaglandin production by indomethacin increased the AQP2 abundance, showing that the dDAVP-stimulated AQP2 abundance is likely reduced due to the effects of endogenously produced prostaglandins. The major prostaglandins produced in mpkCCD cells are PGE_2_ and PGF_2α_. The dDAVP stimulation significantly increased both the production of PGE_2_ and PGD_2_, while levels of PGF_2α_ were decreased. In agreement with these findings, it has been shown that AVP stimulates the PGE_2_ synthesis in isolated collecting ducts (Schlondorff et al., 1985; Bonvalet et al., 1987).

### In MpkCCD Cells, dDAVP-Induced Changes in PGE_2_ Receptor Expression and Activation Explain the Different Effects of PGE_2_ on AQP2 Abundance in the Presence or Absence of AVP

**Figure 6:**
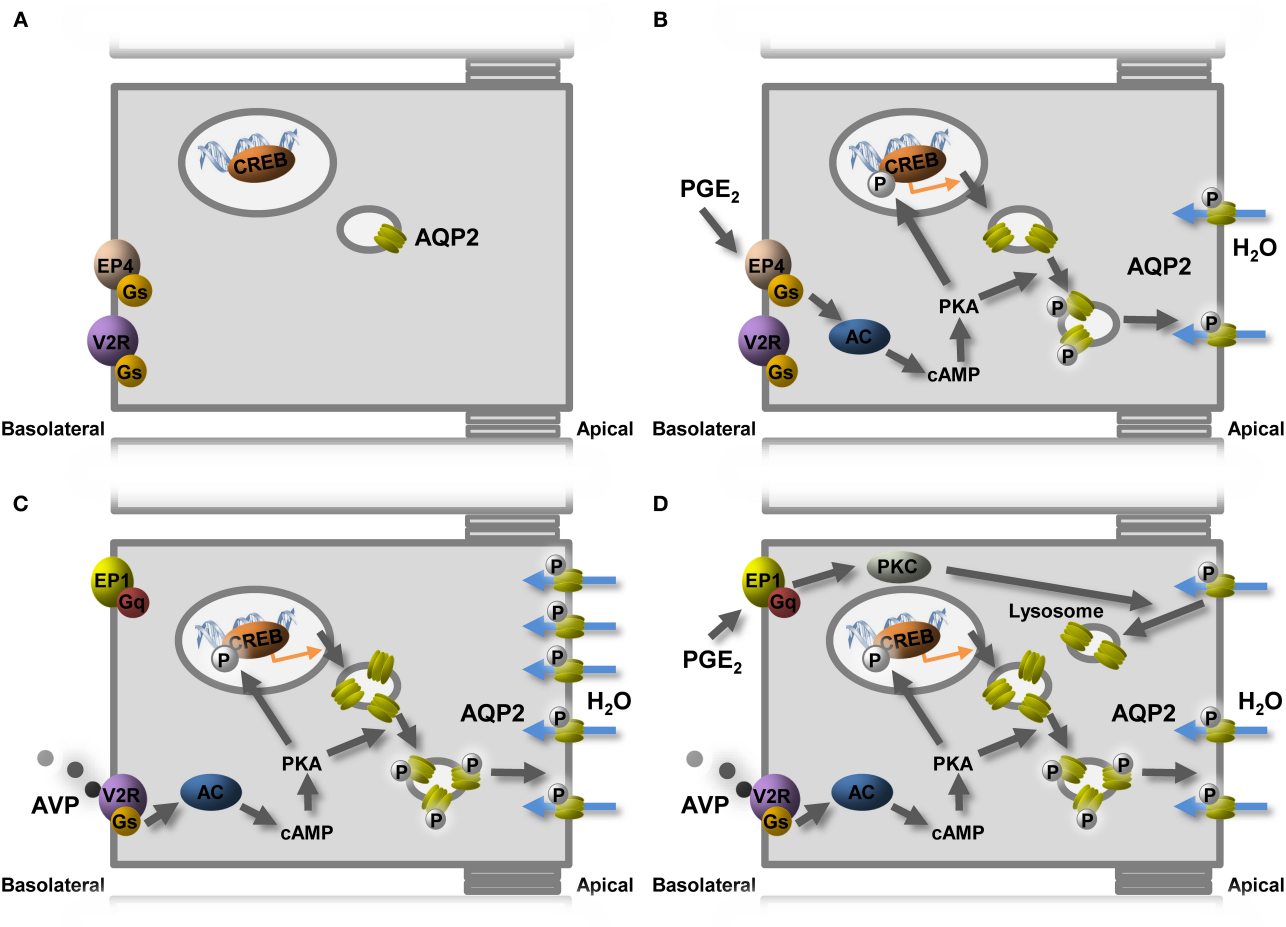
Model of PGE_2_-mediated regulation of AQP2-mediated water reabsorption. **(A)** In the absence of AVP, the AQP2 expression is low and present in intracellular vesicles. **(B)** PGE_2_ stimulates water reabsorption by binding to the EP4 receptor, coupling to the Gs protein, leading to cAMP generation, followed by AQP2 transcription and translocation. **(C)** AVP increases the expression of AQP2 but also induces the expression of the AVP-counteracting EP1 receptor and reduces EP4. **(D)** In the presence of AVP, PGE_2_ decreases the AQP2 expression by stimulating EP1. Indicated are AC, adenylate cyclase; AQP2, aquaporin-2; AVP, vasopressin; cAMP, cyclic adenosine monophosphate; PKA, protein kinase A; PKC, protein kinase C; V2R, vasopressin V2 receptor.

Consistent with previous studies, the PGE_2_ receptors expressed in mpkCCD_c14_ cells are EP1 and EP4 (Olesen et al., 2016). Our experiments using receptor antagonists and agonists show that it is the EP4 receptor that is involved in the stimulatory effect of PGE_2_ on the AQP2 expression in mpkCCD cells. The EP4 receptor can couple to Gs-stimulated cAMP generation, thereby activating the same pathway as AVP. Incubation with dDAVP increased the expression of the EP1 receptor in mpkCCD cells but decreased the expression of the EP4 receptor. Additionally, our experiments showed that the activation of EP1 is the pathway by which PGE_2_ inhibits the dDAVP-induced AQP2 expression in mpkCCD cells (Figure 6). The EP1 receptors can couple to Gq and increase cytosolic Ca^2+^ and activate protein kinase C (PKC; Funk et al., 1993; Watabe et al., 1993). In microperfused collecting ducts, the inhibitory effect of PGE_2_ on AVP-stimulated water permeability was dependent on the activity of PKC (Hebert et al., 1990; Nadler et al., 1992). PKC activation also promotes AQP2 endocytosis, similar to PGE_2_ (Zelenina et al., 2000; Van Balkom et al., 2002; Nejsum et al., 2005), and increases AQP2 ubiquitination, leading to lysosomal degradation (Kamsteeg et al., 2006). This suggests that the EP1 activation will decrease the AQP2 abundance by lysosomal degradation (Figure 6).

The expression of the FP receptor was increased by dDAVP incubation in mpkCCD cells. As the activation of the FP receptor inhibits water reabsorption, the increase in the FP expression might be a compensatory mechanism to counteract AVP stimulation, similar to the increase in the EP1 expression.

As no DP receptor was detected in mpkCCD_c14_ cells, the role of the dDAVP-stimulated increase in the PGD_2_ production after dDAVP incubation is unclear. However, PGD_2_ has been shown to bind to the FP receptor with an affinity close to that for the DP receptor, indicating that PGD_2_ may act on the FP receptor (Kiriyama et al., 1997). The increase in PGD_2_ might counteract the dDAVP-induced increase in the AQP2 expression, although levels are low compared with the PGE_2_ and PGF_2α_ production.

While the TP receptor is expressed in mpkCCD_c14_ cells, the expression of the IP receptor is inconclusive. Both thromboxane and PGI_2_ were produced in very low amounts in mpkCCD_c14_ cells, and the production was not affected by dDAVP. Whether these prostanoids have any role in water reabsorption remains unclear.

### Relation of the MpkCCD Cell System to the *in vivo* Situation

A limitation of our study is that all experiments are performed in mpkCCD cells. However, a problem with *in vivo* studies investigating the effect of prostaglandins on the collecting duct is that these studies are complicated by the effect of prostaglandins on AVP release and on medullary osmolality, both of which will influence the AQP2 expression (Yamamoto et al., 1976; Stoff et al., 1981; Hasler et al., 2005). To study the effect of prostaglandins directly on principal cells, experiments were performed in mpkCCD cells, shown to display the essential functionalities characteristic of principal cells like the AVP-regulated AQP2 expression and aldosterone-mediated sodium transport *via* the epithelial sodium channel (Bens et al., 1999; Hasler et al., 2002).

The major prostaglandins produced in our cell system were PGE_2_ and PGF_2α_, which is in agreement with *in vivo* findings, showing that PGE_2_ is the most abundant prostanoid in both the renal cortex and medulla, followed by PGI_2_ and PGF_2α_ (Qi et al., 2006). The synthases involved in the production of PGD_2_, PGE_2_, and PGF_2α_ are detected in the nephron (Vitzthum et al., 2002; Sakurai et al., 2005), where the production of PGE_2_ and PGF_2α_ has been shown to occur mainly in the collecting ducts (Farman et al., 1987). Neither PGI synthase nor thromboxane synthase mRNA is detected in any tubular structure (Vitzthum et al., 2002).

The effects of prostaglandins on the AQP2 expression are conferred by PG receptors. In mpkCCD_c14_ cells, EP1, EP4, and FP receptors are found, in agreement with expression in the collecting duct (Breyer et al., 1998; Saito et al., 2003).

In line with our data showing the role of EP4 in the stimulatory effect of PGE_2_ on AQP2, a study by Gao et al. demonstrates that disruption of EP4 in the collecting duct impaired the urinary concentration by decreasing the AQP2 abundance and apical membrane targeting, providing evidence that EP4 can regulate the urine concentration *in vivo* (Gao et al., 2015). In addition, a selective EP4 agonist has been shown to increase the urine osmolality, decrease the urine volume, and increase the AQP2 expression in a mouse model for congenital NDI (Li et al., 2009).

In agreement with our findings that the activation of the EP1 receptor decreases the AVP-induced AQP2 expression, the stimulation of the EP1 receptor has been shown to decrease the vasotocin-induced osmotic water permeability of the frog urinary bladder, a model system of the collecting duct (Bachteeva et al., 2007). In addition, EP1-knockout mice have a urinary concentrating defect (Kennedy et al., 2007), and recent studies show that PGE_2_ does not decrease AVP-mediated water transport in isolated collecting ducts of these mice (Nasrallah et al., 2018). Taken together with the present data, this suggests that EP1 conveys both acute and long-term modulation of the V2R activity.

Furthermore, TP and IP receptors are mainly localized in the glomerulus and vasculature, respectively, but have also been located in the collecting duct (Takahashi et al., 1996; Komhoff et al., 1998), in agreement with the expression seen in mpkCCD_c14_ cells. Based on our mpkCCD data, however, we anticipated that the IP receptor does not have a major impact on the principal cell AQP2 expression in the presence or absence of AVP.

None of the receptors DP, EP2, and EP3 seems to be expressed in mpkCCD_c14_ cells. While DP is also not expressed in the kidney, the presence of EP2 along the nephron is a matter of considerable debate (Breyer and Breyer, 2000; Olesen and Fenton, 2013). However, a previous study has shown that functionally, the collecting duct can respond to the stimulation of the EP2 receptor (Olesen et al., 2011).

The inhibitory effects of PGE_2_ on AVP-induced water reabsorption have, besides *via* the activation of EP1, also been suggested to occur through the activation of EP3 (Hebert et al., 1993; Fleming et al., 1998). Our cell model does not express the EP3 receptor, which was found *in vivo* by the *in situ* hybridization and RT-PCR on microdissected tubules to be expressed in the collecting duct (Breyer et al., 1998). However, a study using single-cell RNA-Seq of intercalated and principal cells from the mouse kidney demonstrated that EP3 was selectively expressed in collecting duct-intercalated cells, while EP1 and EP4 were expressed in the principal cells (Chen et al., 2017). In addition, EP3-knockout mice exhibit a similar urine-concentrating ability during basal conditions as well as in response to AVP compared with wild-type mice, arguing against a role of EP3 in the AQP2 regulation (Fleming et al., 1998). The exact role of the EP3 receptor in the AQP2 regulation needs further investigation.

### Central Mechanism for the Differential Effect of PGE_2_ on AQP2 Expression

It is interesting to note that, while dDAVP increases the PGE_2_ production and release, the mRNA expression of the EP4 receptor is reduced, whereas that of the EP1 receptor is increased. As both receptors are bound and activated by PGE_2_, these data suggest that it is not the agonist *per se* that determines the expression level of the receptors. Instead, our data indicate that the signaling cascade that is mainly activated exerts a negative feedback regulation on receptors stimulating the same pathway and a positive feedback on receptors activating an opposite pathway: dDAVP increases the cAMP-AQP2 pathway, which can be stimulated by EP4, whereas EPl activates a pathway that leads to a decreased AQP2 expression and water permeability.

The same antagonizing mechanism can be seen in response to endothelin, which counteracts the AVP-mediated water permeability (Edwards et al., 1993), and at the same time, leads to an increased expression of the vasopressin V2 receptor in the inner medullary collecting duct of the rat (Sonntag et al., 2004). Similar to this antagonizing mechanism, dDAVP increases the mRNA levels of the purinergic receptor subunit P2Y_2_ in mpkCCD cells and targets the subunits P2Y_2_ and P2X_2_ to the plasma membrane, where the activation of these receptors leads to the AQP2 internalization and a decrease in the water permeability (Wildman et al., 2009). A similar mechanism can be seen with the hormone angiotensin II, which increases renal proximal sodium reabsorption but at the same time increases expression of the D4 dopamine receptor in renal proximal tubule cells, which activation will decrease sodium reabsorption, thereby counteracting the direct effect of angiotensin II (Tang et al., 2017).

In conclusion, our study shows that in mpkCCD_c14_ cells, both PGE_2_ and PGF_2α_ decrease the dDAVP-stimulated AQP2 abundance, while in the absence of dDAVP, PGE_2_ increases AQP2 levels. Furthermore, our study suggests that EP4 mediates the PGE_2_-induced increase in the AQP2 abundance in the absence of dDAVP, while the PGE_2_-mediated decrease in the AQP2 abundance in the presence of dDAVP is likely mediated *via* EP1. This paradoxical difference in response to PGE_2_ is likely explained by the different receptor subtype expression induced by the dDAVP treatment, leading to an increase in EP1 and a decrease in EP4.

Based on our data above that a negative feedback is mediated by the signaling pathways activated instead of the agonist, we hypothesized that *in vivo* AVP increases, besides AQP2, the expression of EP1 and decreases the expression of EP4 receptors. Consequently, in conditions with the increased PGE_2_ release, such as with lithium-NDI or bilateral uteral obstruction, the AVP-induced AQP2 expression would be reduced *via* the activation of these EP1 receptors.

## Data Availability Statement

The raw data supporting the conclusions of this article will be made available by the authors, without undue reservation.

## Author Contributions

MK, JW, RF, and PD designed experiments. MK, MB, HS, EO, and CC performed experiments. MK and PD wrote manuscript. All authors approved the final manuscript.

## Funding

PD is a recipient of VICI grant 865.07.0h02 of the Netherlands Organization for Scientific research (NWO). This work was supported by grants from NWO (VICI grant 865.07.002) and RUNMC (2004.55) to PD and grants from the Independent Research Fund Denmark (Project No. 1333-00279 and 1331-00738B) and the Aarhus University Research Foundation to MK. RF was funded by the Independent Research Fund Denmark (Project No. 1026-00063B) and the Novo Nordisk Foundation.

## Conflict of Interest

The authors declare that the research was conducted in the absence of any commercial or financial relationships that could be construed as a potential conflict of interest.

## Publisher's Note

All claims expressed in this article are solely those of the authors and do not necessarily represent those of their affiliated organizations, or those of the publisher, the editors and the reviewers. Any product that may be evaluated in this article, or claim that may be made by its manufacturer, is not guaranteed or endorsed by the publisher.
